# A multiplicative effect of Education and Wealth associated with HIV-related knowledge and attitudes among Ghanaian women

**DOI:** 10.1186/s12889-023-16311-5

**Published:** 2023-07-20

**Authors:** Jonathan Aseye Nutakor, Lulin Zhou, Ebenezer Larnyo, Stephen Addai-Dansoh, Yupeng Cui, Jonathan Kissi, Nana Ama Asi Danso, Alexander Kwame Gavu

**Affiliations:** 1 School of Management, Jiangsu University, Zhenjiang, Jiangsu Province China; 2 Center for Black Studies Research, University of California, Santa Barbara, CA United States of America; 3 Department of Health Information Management, College of Health and Allied Sciences, School of Allied Health Sciences, University of Cape Coast, Cape Coast, Ghana; 4 School of Public Health, University of Ghana, Accra, Ghana; 5 Department of Educational Administration, University of Saskatchewan, Saskatoon, SK Canada

**Keywords:** HIV/AIDS, Knowledge, Attitude, socioeconomic status, Women

## Abstract

**Background:**

Knowledge and attitudes regarding HIV play a crucial role in prevention and control efforts. Understanding the factors influencing HIV-related knowledge and attitudes is essential for formulating effective interventions and policies. This study aims to investigate the possibility of an interaction between education and wealth in influencing HIV-related knowledge and attitudes among women in Ghana.

**Methods:**

Cross-sectional data from the Ghana Multiple Indicator Cluster Survey (MICS), a nationally representative sample, were analyzed. Statistical summaries were computed using place of residence, marital status, education level, wealth index quintile, use of insurance, functional difficulties, and exposure to modern media. Furthermore, a three-model Logistic regression analysis was conducted; Model 1 with main effects only, Model 2 with the interaction between education and wealth, and Model 3 with additional covariates. To account for the complexity of the survey data, the svyset command was executed in STATA.

**Results:**

Although most interaction terms between wealth index quintiles and education levels did not show statistical significance, a few exceptions were observed. Notably, women with primary education in the second, middle, and fourth wealth quintiles, along with those with secondary education in the second wealth quintile, exhibited a negative significant association with HIV-related attitude level. However, no significant associations were found between other factors, including age, place of residence, marital status, and health insurance, and HIV-related attitude. The study also found significant associations between socioeconomic variables and HIV-related knowledge. There was a significant positive association between higher levels of education and HIV-related knowledge level. Women in wealthier quintiles had a significant positive association with HIV-related knowledge level. Factors such as place of residence and media exposure, including radio and television were also observed to be associated with HIV-related knowledge level.

**Conclusions:**

This study highlights the importance of socioeconomic status and media exposure in shaping HIV-related knowledge and attitudes among women in Ghana. Policy interventions should focus on reducing socioeconomic disparities, ensuring equitable access to education and healthcare services, and utilizing media platforms for effective HIV information dissemination.

## Background

Human immunodeficiency virus (HIV) is a major public health problem in Ghana [1]. On average, 350,000 Ghanaians were infected with HIV in 2021 [2]. Women are disproportionately affected by the HIV epidemic, accounting for more than half of the approximately 19,000 new infections in Ghana each year [3, 4]. In recent years, this trend has not changed. There are significant variations in the HIV prevalence and incidence rates across different population groups in Sub-Saharan Africa, with women from poorer socioeconomic backgrounds having a higher risk of infection [5]. The overall prevalence of HIV among adults in Ghana has decreased from 2.1% to 2012 to 1.7% in 2018, which is lower than the global average of 0.8% [6]. However, the HIV prevalence among women in Ghana is higher than that of males [6]. The disparities in HIV prevalence among women in Ghana can be linked to a number of causes, including but not limited to poverty, limited access to education and health services, gender inequality, and cultural norms that continue to perpetuate stigma and discrimination.

In spite of the difficulties, Ghana has made great progress in its fight against HIV and AIDS [7]. The country has instituted numerous initiatives for the prevention, treatment, and care of those living with HIV, including providing antiretroviral medicine at no cost to those who are HIV-positive [8]. In addition, Ghana has made substantial headway toward accomplishing the 90-90-90 goals established by the Joint United Nations Programme on HIV/AIDS (UNAIDS) [6]. As of the year 2020, 87% of HIV positive people living in Ghana were aware of their status, 77% were receiving antiretroviral treatment, and 71% had achieved viral suppression [6]. State and non-state actors in Ghana have put in place a number of programs and policies to help women learn more about HIV and change how they feel about it [1]. Education and awareness programs, economic empowerment programs, access to healthcare, advocacy and community mobilization are some of the projects that fall under this category [1]. For instance, the Ghana AIDS Commission, in conjunction with a variety of other stakeholders, has launched a number of education and awareness programs with the goals of expanding participants’ understanding of HIV/AIDS and decreasing the stigma associated with the condition [9]. Despite the efforts of the government of Ghana and international organizations to reduce the number of new HIV infections and increase access to care and treatment, HIV-related stigma and discrimination continue to be major obstacles in the way of achieving universal access to prevention, treatment, care, and support services [10].

It has been demonstrated that a person’s socioeconomic status (SES) is a primary factor in determining health outcomes [11–13], including HIV-related knowledge, attitudes, and behaviors [14]. A person’s socioeconomic status (SES) is a multidimensional concept that relates to their economic, social, and educational standing [15]. It takes into account things like a person’s income, education level, occupation, and social standing [15]. Studies have shown that women from lower SES backgrounds may have poorer levels of HIV-related knowledge and more unfavorable attitudes toward people living with HIV/AIDS compared to those from higher SES backgrounds [16].

In addition, there are differences between Ghana and the global targets, most notably in the area of HIV-related knowledge and attitudes among women based on their socioeconomic status. In this regard, Ghana is falling behind. The Ghana Demographic and Health Survey (GDHS) found that women from the wealthiest households were more likely to know a lot about HIV than women from the poorest households [16]. In a similar vein, the survey discovered that women who came from the wealthiest homes were more likely to have a positive attitude towards people living with HIV than women who came from the poorest households [16]. These differences underline the necessity of focused interventions to address the social determinants of HIV among women in Ghana [17].

Prior research, conducted by Nketiah-Amponsah et al. (2018) and Appiah et al. (2022), has shed light on the individual impacts of education and wealth on HIV-related knowledge and attitudes among women in Ghana [18, 19]. Research has indicated a correlation between increased levels of education and greater wealth with enhanced knowledge about HIV and more favorable attitudes towards the virus [18, 19]. Nevertheless, an area that has received limited attention is the possible interaction between educational attainment and wealth in influencing the HIV outcomes of women in Ghana [7, 18, 20].

The main objective of this study is to investigate the possibility of an interaction between education and wealth in influencing the knowledge and attitudes towards HIV among women in Ghana, going beyond its main effects. The purpose of this study is to offer a more nuanced understanding of how HIV outcomes are affected by socioeconomic status within this population group, by taking into account the combined influence of these socioeconomic factors. Understanding the interaction between education and wealth holds significant importance for several reasons. Education is an important variable in determining the acquisition of knowledge and critical thinking skills, which empowers individuals to make informed decisions and develop favorable attitudes towards HIV [21]. In order to lower the risk of infection and encourage healthy behaviors, it gives people the knowledge they need regarding HIV prevention, transmission, and treatment [22–24].

On the other hand, wealth refers to the ability to obtain and utilize various assets and opportunities. There is a positive correlation between a woman’s level of wealth and her ability to access healthcare services, such as HIV testing, counseling, and treatment [18, 19]. The attainment of economic stability can equip individuals with the resources to access reliable information from diverse sources, including educational programs, community initiatives, and healthcare professionals [25]. Hence, it is imperative to understand the interaction between education and wealth in influencing knowledge and attitudes towards HIV, in order to mitigate socioeconomic inequalities and devise tailored interventions that cater to diverse populations.

By investigating the interaction between education and wealth in relation to HIV knowledge and attitudes among Ghanaian women, this study aims to contribute to the existing literature and provide policymakers, healthcare providers, and other stakeholders with evidence-based insights. We can design more effective methods to promote HIV-related information, attitudes, and behaviors among Ghanaian women by studying the complex interplay of socioeconomic determinants, ultimately working to reduce the country’s HIV/AIDS burden.

## Methods

**Fig. 1:**
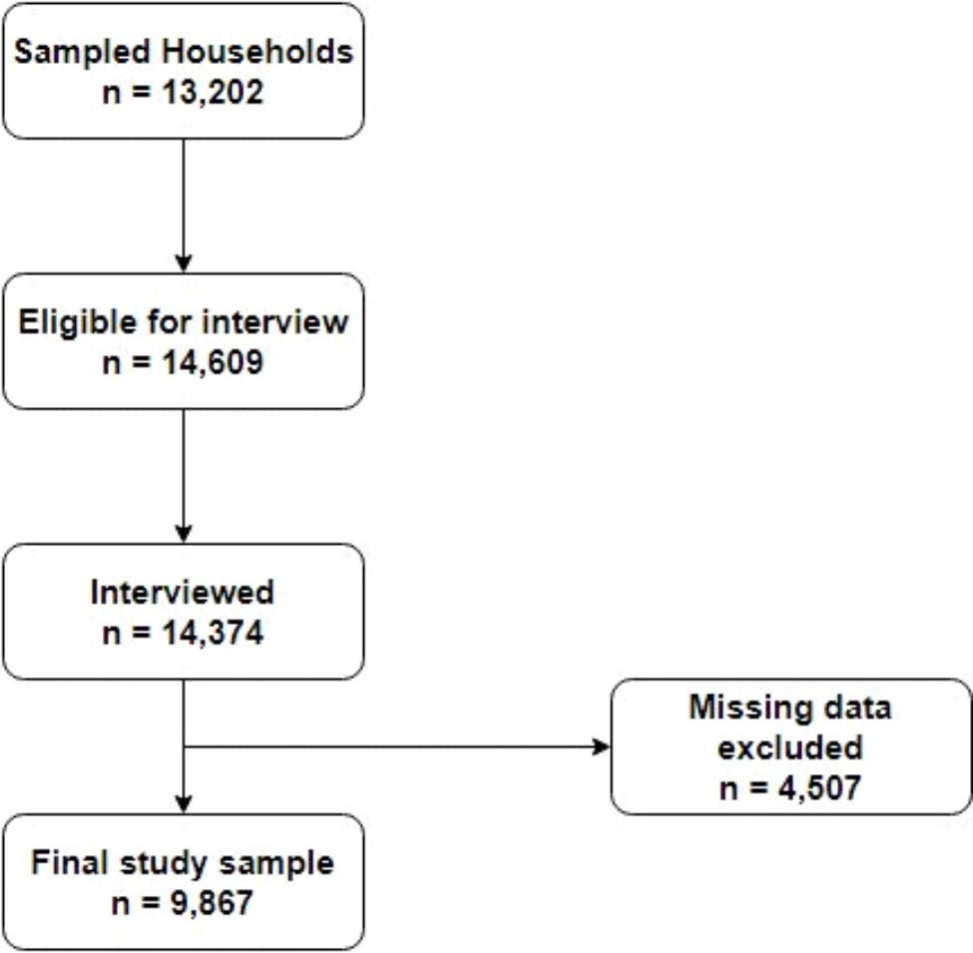
Flowchart illustrating the procedure for including and excluding participants

This study was carried out using cross-sectional data from the Ghana Multiple Indicator Cluster Survey (MICS). MICS is a sample that is nationally representative. The Ghana Statistical Service, along with the Ghana Health Service, the Ghana Education Service, Ministries of Health, Education, Sanitation and Water Resources, and Gender, Children, and Social Protection, worked together to gather the data for this study. In addition, the United Nations International Children’s Emergency Fund (UNICEF) and Catalyst Fund offered expert assistance, and the Korea International Cooperation Agency (KOICA), United Nations Development Programme (UNDP), UNICEF, USAID, and the World Bank provided financial assistance [26]. The Ghana Health Service Ethics Review Committee approved the protocols for the study. Before administering questionnaires to any adult participant, verbal consent was sought. The consent of individuals between the ages of 15 and 17 was sought from their guardians or parents. The participants were assured of their free will to withdraw from the interview at any time, as well as the anonymity and privacy of their responses. Ghanaians between the ages of 15 and 49 were the focus of the study. To choose the participants, a two-step sampling method was used. To begin, 660 enumeration areas/clusters were chosen. The selection of 13,202 households was the next step in the recruitment and selection process (Fig. 1). This study was open to women in the selected households between the ages of 15 and 49. Precisely 14,609 women were found in the selected households, and a total of 14,374 of those women agreed to be interviewed, which is equivalent to a response rate of 98.4%.

### Outcome variables

This study’s outcome variables include HIV-related knowledge and attitudes toward HIV-positive people. The responses to HIV-related knowledge questions were used to build the binary outcome HIV knowledge variable. The responses ‘do not know,‘ ‘not sure,‘ and ‘it depends’ were classified as ‘no’. The right answer was coded as one (1) and the wrong answer as zero (0). The rationale behind reclassifying these ambiguous responses as ‘no’ is to ensure consistency and reduce potential bias in the analysis. In the context of HIV-related knowledge, responses like ‘do not know,‘ ‘not sure,‘ and ‘it depends’ indicate a lack of definitive knowledge or uncertainty. By classifying these responses as ‘no,‘ we avoid artificially inflating the proportion of correct answers and maintain a clear distinction between those who possess accurate HIV-related knowledge and those who do not [27]. The range of overall HIV-related knowledge scores was from 1 to 11. The HIV knowledge threshold was set at > 7 for “high knowledge” and ≤ 7 for “poor knowledge.“ This study recognizes that there is no universally agreed-upon cutoff for defining “high knowledge” regarding HIV. However, previous studies have used various thresholds or cutoff points based on the distribution of knowledge scores in their respective datasets [27, 28]. The seven attitude questions were used to produce the binary HIV-related attitude variable. Responses that said “yes” were coded as “1,“ and responses that said “no” were coded as “0”. After adding up the scores from seven questions, the “attitude toward HIV” variable was put into two groups: “positive attitude” (> 2) and “negative attitude” (≤ 2), again based on what the experts suggested and the distribution of attitude scores [27, 28].

### Independent variables

The participants’ educational attainment was classified into five categories, namely pre-primary education or none, primary education, junior secondary education, senior secondary education, and higher education. Similarly, household income was divided into five categories. These income groups ranged from the poorest to the richest. The wealth index was computed based on the participant’s economic standing, as determined by household information regarding the ownership of consumer goods and household characteristics. Sociodemographic variables included age [15–19, 20–24, 25–29, 30–34, 35–39, 40–44, and 45–49], marital status (currently married, formerly married, and never married), where a person lived (urban/rural), whether they had health insurance, functional difficulties, and how much they were exposed to modern media.

### Statistical analysis

The data was analyzed with STATA SE 14.2 (Stata Corp., College Station, TX, USA). The following categories of information were used to compile statistical summaries: age, place of residence, marital status, level of education, wealth index quintile, use of insurance, functional difficulties, and exposure to modern media. In order to prevent analytical errors [29], we used the svyset command in STATA in accounting for the complex survey design. The study utilized logistic regression analysis to explore the interactions between education and wealth index quintile in relation to HIV-related knowledge and attitudes. The study also examined the impact of socioeconomic status variables and other sociodemographic factors on individuals’ knowledge about HIV and their attitudes towards people with HIV. The analysis involved three models. Model 1 estimates main effects only. Model 2 extends by adding the interaction between education and wealth and Model 3 extends by adding the covariates. Equations for the three models are:

- Model 1 (Main Effects Only): HIV_knowledge/attitude = β0 + β1 * education + β2 * wealth + ε.
- Model 2 (Including Interaction): HIV_knowledge/attitude = β0 + β1 * education + β2 * wealth + β3 * (education * wealth) + ε.
- Model 3 (Adding Covariates): HIV_knowledge/attitude = β0 + β1 * education + β2 * wealth + β3 * (education * wealth) + β4 * age + β5 * residence + β6 * marital status + β7 * health insurance + β8 * functional difficulties + β9 * frequency of reading newspaper + β10 * frequency of listening to radio + β11 * frequency of watching TV + β12 * ever used computer + β13 * ever used internet + ε.

Where β0 represents the intercept or constant term, β1 the coefficient associated with the variable education, β2 the coefficient associated with the variable wealth, β3 the coefficient associated with the interaction term (education * wealth), β4 to β13 representing the coefficient associated with the various covariates, and ε the error term. The significance level was set at 0.05, and the confidence interval was set at 95%.

## Results

Table 1 provides a description of the women participants of this study. The study consists of 9867 women. Most of the women were between 20 and 24 years old (23.15%). Relative to those living in urban areas, 50.18% resided in rural areas. In terms of marital status, the majority of respondents (63.03%) were married. Surprisingly, most of the respondents to the survey (22.36%) had only completed pre-primary school or had no education at all. The respondents were divided into five quintiles based on their wealth. Most of the respondents to the survey (21.77%) were in the richest group. In addition, the majority of respondents (57.13%) reported having insurance and having no functional difficulties (91.04%). The study also assessed respondents’ exposure to modern media. In terms of the frequency with which they read newspapers or magazines, only 0.76% of respondents read daily, compared to 90.09% who do not read at all. When asked how often they listen to the radio, most of the respondents (31.68%) said they never listen. When asked how often they watched television (TV), most of the respondents said that they did so almost every day. The respondents were asked if they have ever used a computer or the internet. About 87% of respondents have never used a computer, and 83.28% have never used the internet. On the basis of other descriptive statistics, the respondent’s knowledge and attitude towards HIV positive individuals were evaluated. Among a total of 9867 participants, we found that 8460 had a good Knowledge of HIV. On the other hand, it was found that only 34 of the respondents had a positive attitude towards HIV positive individuals.

**Table 1:** Socio-demographic characteristics of study participants classified by HIV knowledge level and attitude toward HIV-positive individuals

Variable		Frequency	Percentage	HIV knowledge level		HIV attitude level	
		n = 9867		High (8460)	Low (1407)	Positive (34)	Negative (9833)
**Age group**	15–19	838	8.49	741	97	4	834
	20–24	2284	23.15	2,026	258	9	2275
	25–29	1607	16.29	1378	229	4	1603
	30–34	1367	13.85	1178	189	4	1363
	35–39	1393	14.12	1128	265	6	1387
	40–44	1273	12.9	1066	207	3	1270
	45–49	1105	11.2	943	162	4	1101
**Residence**	Rural	4951	50.18	4056	895	26	4925
	Urban	4916	49.82	4404	512	8	4908
**Marital status**	Currently married	6219	63.03	5220	999	23	6196
	Formerly married	1040	10.54	870	170	3	1037
	Never married	2608	26.43	2370	238	8	2600
**Education Level**	Pre-primary or none	2206	22.36	1778	428	12	2194
	Primary	1622	16.44	1314	308	11	1611
	JSS/JHS/Middle	3580	36.28	3063	517	8	3572
	SSS/SHS/ Secondary	1969	19.96	1828	141	3	1966
	Higher	490	4.97	477	13	0	490
**Wealth index quintile**	Poorest	2074	21.02	1681	393	19	2055
	Second	1682	17.05	1367	315	8	1674
	Middle	1933	19.59	1631	302	3	1930
	Fourth	2030	20.57	1796	234	4	2026
	Richest	2148	21.77	1985	163	0	2148
**Health insurance**	Without insurance	4230	42.87	3554	676	20	4210
	With insurance	5637	57.13	4906	731	14	5623
**Functional difficulties**	Has functional difficulty	884	8.96	750	134	4	880
	Has no functional difficulty	8983	91.04	7710	1273	30	8953
**Frequency of reading newspaper or magazine**	Not at all	8889	90.09	7536	1353	33	8856
	Less than once a week	543	5.5	511	32	1	542
	At least once a week	360	3.65	342	18	0	360
	Almost everyday	75	0.76	71	4	0	75
**Frequency of listening to the radio**	Not at all	3126	31.68	2644	482	19	3107
	Less than once a week	1635	16.57	1369	266	5	1630
	At least once a week	2092	21.2	1845	247	6	2086
	Almost everyday	3014	30.55	2602	412	4	3010
**Frequency of watching TV**	Not at all	2957	29.97	2453	504	20	2937
	Less than once a week	1015	10.29	837	178	5	1010
	At least once a week	1586	16.07	1384	202	2	1584
	Almost everyday	4309	43.67	3786	523	7	4302
**Ever used a computer or a tablet**	No	8556	86.71	7235	1321	33	8523
	Yes	1,311	13.29	1225	86	1	1310
**Ever used internet**	No	8217	83.28	6907	1310	33	8184
	Yes	1650	16.72	1553	97	1	1649

Table 2 presents results across three distinct models (Model 1, Model 2, and Model 3) that pertain to the level of HIV knowledge. According to Model 1, there is a significant positive association between JSS/JHS/Middle, SSS/SHS/Secondary, and higher education, and HIV knowledge level, compared to individuals with no formal education or pre-primary education [Coef. = 0.03, 95% (CI: 0.01–0.50)], [Coef. = 0.09, 95% (CI: 0.07–0.11)], and [Coef. = 0.12, 95% (CI: 0.08–0.15)]. In Model 2, only the positive association between JSS/JHS/Middle and SSS/SHS/Secondary education level, and HIV knowledge level remained significant [Coef. = 0.06, 95% (CI: 0.03–0.10)] and [Coef. = 0.21, 95% (CI: -0.01-0.43)]. Similarly, in Model 3, only the positive association between JSS/JHS/Middle and SSS/SHS/Secondary education, and HIV knowledge level remained significant [Coef. = 0.05, 95% (CI: 0.01–0.09)] and [Coef. = 0.09, 95% (CI: 0.03–0.15)]. In reference to the Wealth Index Quintile, it was observed across all three models that there is a significant positive association between individuals belonging to the fourth and richest quintiles and HIV knowledge level in comparison to those belonging to the poorest wealth quintile [Coef. = 0.04, 95% (CI: 0.02–0.06)] and [Coef. = 0.06, 95% (CI: 0.04–0.08)] in Model 1, [Coef. = 0.07, 95% (CI: 0.02–0.12)] and [Coef. = 0.09, 95% (CI: 0.03–0.16)] in Model 2, and [Coef. = 0.06, 95% (CI: 0.01–0.11)] and [Coef. = 0.08, 95% (CI: 0.01–0.15)] in Model 3. Regarding the place of residence of the participants, Model 3 shows a significant negative association between women living in urban areas and HIV knowledge level compared to their rural counterparts [Coef. = -0.03, 95% (CI: -0.04 to -0.01)]. Model 3 shows a significant negative association between individuals who listen to the radio less than once a week and HIV knowledge level compared to those who do not listen to the radio at all, with respect to the Frequency of Listening to the Radio [Coef. = -0.02, 95% (CI: -0.04 to -0.01)]. Also, Model 3 shows a significant negative association between individuals who watch TV less than once a week and HIV knowledge level compared to those who abstain from watching TV altogether, in relation to the frequency of watching television [Coef. = -0.02, 95% (CI: -0.04 to -0.01)].

**Table 2:** Associations between HIV knowledge level and education, wealth, and their interactions

Variable	HIV knowledge level		
	Model 1	Model 2	Model 3
	Coef. (95% CI)	Coef. (95% CI)	Coef. (95% CI)
Education Level			
Pre-primary or none	ref	ref	ref
Primary	-0.01 [-0.02; 0.02]	-0.02 [-0.06; 0.01]	-0.02 [-0.06; 0.01]
JSS/JHS/Middle	0.03 [0.01; 0.50] ***	0.06 [0.03; 0.10] ***	0.05 [0.01; 0.09] **
SSS/SHS/ Secondary	0.09 [0.07; 0.11] ***	0.21 [-0.01; 0.43] ***	0.09 [0.03; 0.15] ***
Higher	0.12 [0.08; 0.15] ***	0.21 [-0.01; 0.43]	0.15 [-0.06; 0.38]
Wealth index quintile			
Poorest	ref	ref	ref
Second	-0.01 [-0.03; 0.01]	-0.01 [-0.04; 0.02]	-0.01 [-0.04; 0.03]
Middle	0.01 [-0.01; 0.03]	0.02 [-0.01; 0.06]	0.01 [-0.02; 0.06]
Fourth	0.04 [0.02; 0.06] ***	0.07 [0.02; 0.12] ***	0.06 [0.01; 0.11] **
Richest	0.06 [0.04; 0.08] ***	0.09 [0.03; 0.16] ***	0.08 [0.01; 0.15] **
Interactions			
Education#Wealth			
Primary#Second		0.01 [-0.04; 0.07]	0.01 [-0.04; 0.07]
Primary#Middle		0.02 [-0.04; 0.08]	0.02 [-0.03; 0.09]
Primary#Fourth		0.02 [-0.03; 0.09]	0.03 [-0.03; 0.10]
Primary#Richest		0.03 [-0.05; 1.12]	0.03 [-0.05; 0.12]
JSS/JHS/Middle#Second		-0.02 [-0.07; 0.03]	-0.02 [-0.07; 0.03]
JSS/JHS/Middle#Middle		-0.04 [-0.10; 0.01]	-0.04 [-0.09; 0.01]
JSS/JHS/Middle#Fourth		-0.07 [-0.13; -0.01]	-0.06 [-0.12; -0.01]
JSS/JHS/Middle#Richest		-0.06 [-0.01; 0.01]	-0.06 [-1.13; 0.01]
SSS/SHS/ Secondary#Second		-0.02 [-0.10; 0.05]	-0.02 [-0.10; 0.05]
SSS/SHS/ Secondary#Middle		-0.03 [-0.10; 0.04]	-0.02 [-0.10; 0.04]
SSS/SHS/ Secondary#Fourth		-0.05 [-0.13; 0.02]	-0.06 [-0.13; 0.01]
SSS/SHS/ Secondary#Richest		-0.06 [-0.15; 0.02]	-0.06 [-0.15; 0.02]
Higher#Second		-0.04 [-0.32; 0.23]	-0.02; [-0.29; 0.25]
Higher#Middle		-0.09 [-0.35; 0.16]	-0.06 [-0.32; 0.19]
Higher#Fourth		-0.08 [-0.32; 0.15]	-0.06 [-0.30; 0.16]
Higher#Richest		-0.12 [-0.36; 0.11]	-0.10 [-0.34; 0.12]
Age group			
15–19			Ref
20–24			0.01 [-0.02; 0.03]
25–29			-0.01 [-0.03; 0.02]
30–34			0.01 [-0.02; 0.03]
35–39			-0.03 [-0.06; 0.01]
40–44			-0.01[ -0.04; 0.02]
45–49			0.01 [-0.02; 0.04]
Residence			
Rural			Ref
Urban			-0.03 [-0.04; -0.01] ***
Marital status			
Currently married			Ref
Formerly married			-0.01 [-0.02; 0.01]
Never married			0.01 [-0.01; 0.03]
Health insurance			
Without insurance			Ref
With insurance			-0.01 [-0.02; 0.01]
Functional difficulties			
Has functional difficulty			Ref
Has no functional difficulty			-0.01 [0.03; 0.01]
Frequency of reading newspaper or magazine			
Not at all			Ref
Less than once a week			0.02 [-0.01; 0.05]
At least once a week			0.03 [-0.01; 0.07]
Almost everyday			0.01 [-0.06; 0.09]
Frequency of listening to the radio			
Not at all			Ref
Less than once a week			-0.02 [-0.04; -0.01] *
At least once a week			0.01 [-0.01; 0.02]
Almost everyday			-0.01 [-0.03; 0.01]
Frequency of watching TV			
Not at all			Ref
Less than once a week			-0.02 [-0.04; -0.01] **
At least once a week			-0.01 [-0.03; 0.01]
Almost everyday			-0.01 [-0.03; 0.01]
Ever used a computer or a tablet			
No			Ref
Yes			0.01 [-0.02; 0.02]
Ever used internet			
No			Ref
Yes			-0.02 [-0.04; 0.01]

***p < 0.001; **p < 0.01; *p < 0.05; Coef. - Coefficient; CI – Confidence Interval; # - Interaction

Table 3 presents results across three distinct models (Model 1, Model 2, and Model 3) that pertain to the level of HIV attitude. With regards to the level of education, Model 1 does not exhibit any significant associations between the level of education and the level of attitude towards HIV. According to the findings of Model 2 and Model 3, it was observed that women with primary education shows a significant positive association with HIV attitude compared to those with no formal education or have only completed pre-primary education [Coef. = 0.01, 95% (CI: -0.01 to 0.02)]. No other education levels show significant association with HIV attitude in all three models. In Model 1, it was observed that individuals belonging to the second, middle, fourth, and richest wealth quintiles shows significant negative associations with HIV attitude level as compared to those belonging to the poorest wealth quintile [(Coef. = -0.01, 95% (CI: -0.01 to 0.01)], [(Coef. = -0.01, 95% (CI: -0.01 to 0.01)], [Coef. = -0.01, 95% (CI: -0.02 to 0.01)], and [Coef. = -0.01, 95% (CI: -0.01 to 0.01)]. However, these associations are not consistent across all three models.

The results include the interaction between quintiles of wealth index and levels of education. The findings suggest that, with the exception of a limited number of instances, the interaction terms did not exhibit statistical significance. For example, the interaction of primary education and being in the second quintile Model 1: [Coef. = -0.01, 95% (CI: -0.02, 0.01)]; Model 2: [Coef. = -0.01, 95% (CI -0.02, 0.01)], the interaction of primary education and the middle quintile Model 1: [Coef. = -0.01, 95% (CI -0.02, 0.01)]; Model 2: [Coef. = -0.01, 95% (CI -0.02, 0.01)], the interaction of primary education and being in the fourth quintile Model 1: [Coef. = -0.01, 95% (CI -0.02, 0.01)]; Model 2: [Coef. = -0.01, 95% (CI -0.02, 0.01)], as well as the interaction of SSS/SHS/Secondary and the second quintile Model 1: [Coef. = -0.01, 95% CI -0.02, 0.01)]; Model 2: [Coef. = -0.01, 95% CI -0.03, 0.01)], shows a significant negative association with HIV attitude level. The results also include an analysis of other factors such as age, place of residence, marital status, health insurance, functional difficulties, frequency of reading newspapers or magazines, frequency of listening to the radio, frequency of watching TV, ever using a computer or a tablet, and ever using the internet. Nevertheless, no significant associations were observed between these variables and the level of attitude towards HIV.

**Table 3:** Associations between HIV attitude level and education, wealth, and their interactions

Variable	HIV attitude level		
	Model 1	Model 2	Model 3
	Coef. (95% CI)	Coef. (95% CI)	Coef. (95% CI)
Education Level			
Pre-primary or none	ref	ref	ref
Primary	0.01 [-0.01; 0.02]	0.01 [-0.01; 0.02] ***	0.01 [-0.01; 0.02] ***
JSS/JHS/Middle	-0.01 [-0.01; 0.01]	0.01 [-0.01; 0.01]	0.01 [-0.01; 0.02]
SSS/SHS/ Secondary	-0.01 [-0.01; 0.01]	0.01 [-0.01; 0.02]	0.01 [-0.01; 0.02]
Higher	-0.01 [-0.01; 0.01]	-0.01 [-0.04; 0.03]	-0.01 [-0.04; 0.03]
Wealth index quintile			
Poorest	ref	ref	ref
Second	-0.01 [0.01; -0.01] *	0.01 [-0.01; 0.02]	0.01 [-0.01; 0.02]
Middle	-0.01 [-0.01; -0.01] ***	-0.01 [-0.01; 0.01]	-0.01 [-0.01; 0.01]
Fourth	-0.01 [-0.02; -0.01] ***	-0.01 [-0.01; 0.01]	-0.01 [-0.01; 0.01]
Richest	-0.01 [-0.01; -0.01] ***	-0.01 [0.02; 0.01]	-0.01 [0.02; 0.01]
Interactions			
Education#Wealth			
Primary#Second		-0.01 [-0.02; -0.01] **	-0.01 [-0.02; -0.01] **
Primary#Middle		-0.01 [-0.02; -0.01] **	-0.01 [-0.02; -0.01] **
Primary#Fourth		-0.01 [-0.02; -0.01] *	-0.01 [-0.02; -0.01] *
Primary#Richest		-0.01 [-0.02; 0.01]	-0.01 [-0.03; 0.01]
JSS/JHS/Middle#Second		-0.01 [-0.01; 0.01]	-0.01 [-0.01; 0.01]
JSS/JHS/Middle#Middle		-0.01 [-0.01; 0.01]	-0.01 [-0.01; 0.01]
JSS/JHS/Middle#Fourth		-0.01 [-0.01; 0.01]	-0.01 [-0.01; 0.01]
JSS/JHS/Middle#Richest		-0.01 [-0.01; 0.02]	0.01 [-0.01; 0.02]
SSS/SHS/ Secondary#Second		-0.01 [0.02; 0.01] *	-0.01 [-0.03; -0.01] *
SSS/SHS/ Secondary#Middle		-0.01 [-0.02; 0.01]	-0.01 [-0.02; 0.01]
SSS/SHS/ Secondary#Fourth		-0.01 [-0.02; 0.01]	-0.01 [-0.02; 0.01]
SSS/SHS/ Secondary#Richest		-0.01 [-0.02; 0.01]	-0.01 [-0.02; 0.01]
Higher#Second		-0.01 [-0.04; 0.04]	-0.01 [-0.05; 0.04]
Higher#Middle		0.01 [-0.04; 0.04]	0.01 [-0.04; 0.05]
Higher#Fourth		0.01 [-0.03; 0.04]	0.01 [-0.04; 0.04]
Higher#Richest		0.01 [-0.03; 0.04]	0.01 [-0.04; 0.05]
Age group			
15–19			Ref
20–24			-0.01 [-0.01; 0.01]
25–29			-0.01 [-0.01; 0.01]
30–34			-0.01 [-0.01; 0.01]
35–39			-0.01 [-0.01; 0.01]
40–44			-0.01 [-0.01; 0.01]
45–49			-0.01 [-0.01; 0.01]
Residence			
Rural			Ref
Urban			-0.01 [-0.01; 0.01]
Marital status			
Currently married			Ref
Formerly married			-0.01 [-0.01; 0.01]
Never married			0.01 [-0.01; 0.02]
Health insurance			
Without insurance			Ref
With insurance			0.01 [-0.01; 0.02]
Functional difficulties			
Has functional difficulty			Ref
Has no functional difficulty			-0.01 [-0.01; 0.01]
Frequency of reading newspaper or magazine			
Not at all			Ref
Less than once a week			0.01 [-0.01; 0.02]
At least once a week			-0.01 [-0.01; 0.01]
Almost everyday			-0.01 [-0.01; 0.01]
Frequency of listening to the radio			
Not at all			Ref
Less than once a week			-0.01 [-0.01; 0.01]
At least once a week			-0.01 [-0.01; 0.01]
Almost everyday			-0.01 [-0.01; 0.01]
Frequency of watching TV			
Not at all			Ref
Less than once a week			0.01 [-0.01; 0.02]
At least once a week			-0.01 [-0.01; 0.01]
Almost everyday			-0.01 [-0.01; 0.01]
Ever used a computer or a tablet			
No			Ref
Yes			0.01 [-0.01; 0.02]
Ever used internet			
No			Ref
Yes			0.01 [-0.01; 0.02]

***p < 0.001; **p < 0.01; *p < 0.05; Coef. - Coefficient; CI – Confidence Interval; # - Interaction

## Discussion

The findings found a consistent trend across the models among women with primary education. In particular, the interaction of primary education and being in the second, middle, and fourth wealth quintiles, as well as the interaction of SSS/SHS/Secondary education and the second wealth quintile, were significantly associated with lower levels of poor HIV attitude. Generally, the interaction between quintiles of wealth index and levels of education did not exhibit statistical significance in relation to HIV attitude. However, specific instances demonstrated a notable association between education and wealth quintiles, resulting in lower HIV attitude levels. The findings may suggest a possible disparity in access to resources, knowledge, or cultural factors that influence HIV attitudes among women with primary education. The results suggest a possible disparity in access to resources, knowledge, or cultural factors that influence HIV attitudes among women with primary education [30]. When examining the interactions, it is important to notice that the observed associations are relatively small, as evidenced by the narrow confidence intervals around the odds ratios. However, small associations can have important implications within the context of HIV attitudes and behaviors [31].

To understand why these interactions occur, it is important to consider the role of education and wealth in shaping individuals’ knowledge, perceptions, and attitudes towards HIV. Education may serve as a vital factor in disseminating accurate information about HIV prevention, transmission, and treatment [21]. It equips individuals with the necessary knowledge and awareness to make informed decisions and adopt positive attitudes towards HIV [22–24]. With primary education, individuals gain a basic understanding of HIV-related issues, enabling them to recognize the importance of prevention, testing, and support [22–24]. Moreover, the wealth index represents socioeconomic status, which can influence access to resources and opportunities. Women in higher wealth quintiles often have better access to healthcare services, including HIV testing and counseling, which can enhance their understanding and attitudes towards the virus [25]. Economic stability can also provide them with the means to seek accurate information through various channels, such as educational programs, community initiatives, or professional healthcare providers. Further investigation is required to understand the underlying mechanisms of this interaction. Although there are no consistent interactions between education and wealth for women with secondary education or higher, the findings indicate that there is no significant association or effect between wealth and HIV attitudes among women with higher education.

Comparable studies have reported similar results regarding the interaction between education and wealth in relation to attitudes towards HIV. For instance, one study found that women with primary education who had higher wealth exhibited a more positive attitude towards HIV [27]. However, it is important to acknowledge that there are studies with conflicting results. For example, one study found that higher education served as a protective factor against the detrimental impact of wealth on HIV risk perception among women [32]. Another study found that within the cohort of educated women in India, there was no significant association between increased wealth and more favorable attitudes towards HIV [33]. These divergent outcomes may be due to differences in sample characteristics, cultural settings, and measurement methods [34, 35]. The complex interaction among socioeconomic determinants, educational attainment, and cultural norms may give rise to divergent outcomes among various demographic groups.

It is important to acknowledge that attitudes towards HIV are complex and multifaceted, influenced by a wide range of individual, social, and cultural factors beyond education and wealth. Factors such as cultural beliefs, religious values, gender norms, and community stigmatization may significantly impact HIV attitudes, irrespective of educational or wealth status [36]. Future research should explore these factors comprehensively to gain a deeper understanding of the nuances in shaping HIV attitudes.

The study further investigated the socioeconomic disparities in knowledge and attitudes towards HIV among women in Ghana. The findings revealed that education and wealth were both significantly associated with HIV knowledge. Women with higher levels of education and belonging to the richest wealth quintile had better knowledge of HIV. The study suggests that education may help individuals acquire knowledge and critical thinking skills, which could contribute to their understanding and implementation of preventative measures against HIV [37, 38]. Allocating resources to education and integrating comprehensive sexual health education into academic syllabi may have positive effects on HIV awareness among women in Ghana [25]. The present finding aligns with prior research indicating a positive association between higher socioeconomic status and knowledge and attitude regarding HIV [22, 39, 40]. Additionally, achieving equitable dissemination of HIV knowledge requires addressing socioeconomic disparities and improving access to health information among disadvantaged populations. The study also identified the frequency of media exposure, particularly through radio and television, as a potential tool for improving HIV knowledge dissemination [41–44]. The findings underscore the importance of developing interventions tailored to the specific needs of urban communities and addressing socioeconomic disparities to achieve equitable dissemination of HIV knowledge [45].

The study highlights the importance of education in shaping HIV attitudes and recognizes the influence of wealth as a representation of socioeconomic status on access to healthcare services and the ability to seek accurate information. Although the associations between education, wealth, and HIV attitude were small, the study emphasizes the significance of these associations and their implications for HIV attitudes and behaviors. The findings contribute to understanding the complexities of HIV knowledge and attitudes and provide insights for designing targeted interventions and policies to address disparities and promote equitable dissemination of HIV knowledge among different socioeconomic groups.

The results of this study have important policy implications. They suggest a need to allocate resources towards education and ensure equitable availability of high-quality education to all individuals. Comprehensive sexual health education programs in schools can potentially enhance the HIV knowledge of students. Additionally, it’s important to focus on mitigating socioeconomic disparities and enhancing the accessibility of healthcare services and information for marginalized populations. This involves addressing urban-rural inequities and utilizing media platforms to convey accurate and complete HIV-related information [41].

The study has several strengths. For instance, it utilized a large sample size, which makes it possible to generalize the findings to the population of interest. The study also examined various factors, including level of education, wealth, residence, and media exposure, enabling a comprehensive evaluation of their associations with HIV knowledge and attitudes. Nonetheless, the study has some limitations. For example, it relied on self-reported information, which might be influenced by recall and social desirability bias. Moreover, the cross-sectional design of the study makes it difficult to establish causality. To gain a more thorough understanding of the temporal associations among the variables, longitudinal studies would be more appropriate. Lastly, the results may not be generalizable to other populations or contexts.

## Conclusion

To conclude, this research explored how education and wealth relate to HIV knowledge and attitude among women in Ghana. The results showed that women with primary education had different HIV attitudes based on their wealth quintiles. Education played an important role in shaping HIV attitudes, while wealth represented socioeconomic status and impacted access to healthcare services and information. Although the associations between education, wealth, and HIV attitude were small, they were still significant and highlight the implications for HIV attitudes and behaviors. This study adds to the existing literature by investigating the interaction between education and wealth among women with primary education and recognizing the need for a comprehensive understanding of factors influencing HIV attitudes. Additionally, the study highlights the importance of addressing socioeconomic disparities and using media platforms to disseminate HIV knowledge. Further research, including longitudinal studies, is necessary to establish causality and generalize the findings to other populations and contexts.

## Data Availability

The datasets generated and analyzed during the current study article are available from the Multiple Indicator Cluster Survey (MICS) website (https://mics.unicef.org/surveys).
